# Severe infection including disseminated herpes zoster triggered by subclinical Cushing’s disease: a case report

**DOI:** 10.1186/s12902-021-00757-y

**Published:** 2021-04-27

**Authors:** Yuki Yamauchi, Hiraku Kameda, Kazuno Omori, Michio Tani, Kyu Yong Cho, Akinobu Nakamura, Hideaki Miyoshi, Shinya Tanaka, Tatsuya Atsumi

**Affiliations:** 1 Department of Rheumatology, Endocrinology and Nephrology, Faculty of Medicine, Graduate School of Medicine, Hokkaido University, Sapporo, Japan; 2 Department of Cancer Pathology, Hokkaido University Faculty of Medicine, Sapporo, Japan; 3 Division of Diabetes and Obesity, Faculty of Medicine, Graduate School of Medicine, Hokkaido University, Sapporo, Japan; 4 Institute for Chemical Reaction Design and Discovery (WPI-ICReDD), Hokkaido University, Sapporo, Japan

**Keywords:** Subclinical Cushing’s disease, Disseminated herpes zoster, Infection

## Abstract

**Background:**

Subclinical Cushing’s disease (SCD) is defined by corticotroph adenoma-induced mild hypercortisolism without typical physical features of Cushing’s disease. Infection is an important complication associated with mortality in Cushing’s disease, while no reports on infection in SCD are available. To make clinicians aware of the risk of infection in SCD, we report a case of SCD with disseminated herpes zoster (DHZ) with the mortal outcome.

**Case presentation:**

An 83-year-old Japanese woman was diagnosed with SCD, treated with cabergoline in the outpatient. She was hospitalized for acute pyelonephritis, and her fever gradually resolved with antibiotics. However, herpes zoster appeared on her chest, and the eruptions rapidly spread over the body. She suddenly went into cardiopulmonary arrest and died. Autopsy demonstrated adrenocorticotropic hormone-positive pituitary adenoma, renal abscess, and DHZ.

**Conclusions:**

As immunosuppression caused by SCD may be one of the triggers of severe infection, the patients with SCD should be assessed not only for the metabolic but also for the immunodeficient status.

**Supplementary Information:**

The online version contains supplementary material available at 10.1186/s12902-021-00757-y.

## Background

Cushing’s syndrome (CS) is caused by chronic exposure to excess glucocorticoids. CS is divided between adrenocorticotropic hormone (ACTH)-dependent and ACTH-independent causes [1]. Among ACTH-dependent CS, Cushing’s disease (CD) caused by ACTH-producing pituitary adenoma is most common [1]. In addition to CD, subclinical Cushing’s disease (SCD) has been identified in recent years [2]. SCD is defined by ACTH-induced mild hypercortisolism without typical physical features of CD including moon face, central obesity, buffalo hump, purple striae, thin skin, easy bruising, and proximal myopathy [3]. Even when clinical signs of overt hypercortisolism are not present, mild hypercortisolism is associated with metabolic changes such as hypertension, type 2 diabetes mellitus, dyslipidemia, and obesity, resulting in increased risk of mortality [4, 5]. Infection is one of the major complications related to mortality in the patients with CD [6], while no detailed reports on the risk of infection in the patients with SCD are available. Therefore, the relationship between SCD and infection has remained unknown. We experienced a case in which SCD might be one of the triggers of severe infection. To make clinicians aware of the risk of infection in SCD, we report a case of SCD with disseminated herpes zoster (DHZ) ultimately with the mortal outcome.

## Case presentation

**Fig. 1:**
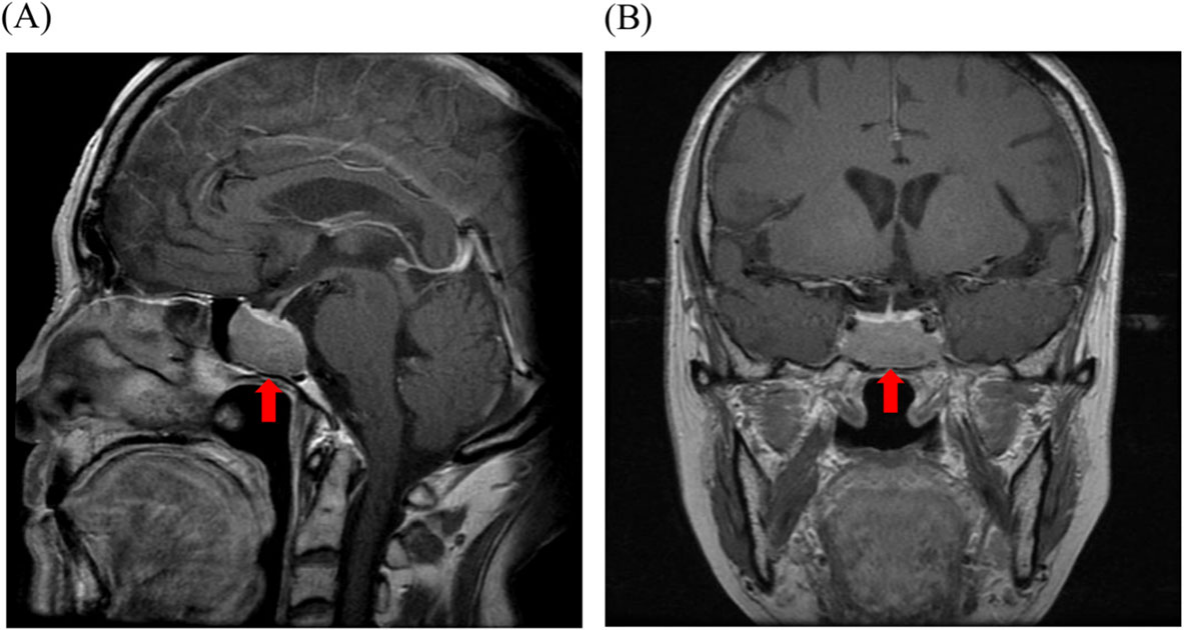
Intracranial magnetic resonance imaging (MRI). **a** Sagittal view of contrast-enhanced T1-weighted MRI. **b** Coronal view of contrast-enhanced T1-weighted MRI. red arrows: pituitary adenoma

An 83-year-old Japanese woman was diagnosed with a 3-cm pituitary tumor by magnetic resonance imaging (MRI) in 2000 (Fig. 1). She was admitted to Hokkaido University Hospital because of general fatigue in 2014. She had no obvious Cushingoid appearance, but her plasma ACTH level and urinary free cortisol level were high. A low-dose (0.5 mg) overnight dexamethasone suppression test (DST) demonstrated incomplete suppression of plasma cortisol levels, and her plasma cortisol levels during night-time sleep remained high. Although her plasma cortisol levels remained high with a high-dose (8 mg) overnight DST, she was diagnosed with SCD because of the presence of a pituitary tumor in MRI (Table 1) [7]. Considering the risk of pituitary surgery at an advanced age and complications including diabetes mellitus, we initiated administration of 0.25 mg of cabergoline per week. The level of ACTH remained high after administration of cabergoline, and we gradually increased the dose of cabergoline from 0.25 mg to 1.5 mg per week in the outpatient. The size of the pituitary tumor did not change every year. Regarding diabetes mellitus, the glycemic control worsened in correlation with the ACTH and cortisol level. She was treated with insulin glargine 12 unit/day, liraglutide 0.9 mg/day, and mitiglinide calcium 30 mg/day and HbA1c level was approximately 8.0 % in the outpatient clinic.

**Table 1:** The results of endocrinological examinations for the diagnosis of SCD in 2014

Basal levels of ACTH and cortisol	
ACTH (pg/mL)	128.1
plasma cortisol (µg/dL)	11.2
urinary cortisol (µg/day)	116.6
Screening tests	
plasma cortisol levels with 0.5 mg overnight DST (µg/dL)	5.5
ACTH levels with 0.5 mg overnight DST (pg/mL)	99.55
plasma cortisol levels during night time sleep (µg/dL)	13.0
Differential diagnosis of Cushing’s disease from EAS	
CRH test	Not performed
(With consideration for the risk of pituitary apoplexy)	
plasma cortisol levels with 8 mg overnight DST (µg/dL)	7.1
ACTH levels with 8 mg overnight DST (pg/mL)	70.19

*ACTH* adrenocorticotropic hormone, *CRH* corticotropin releasing hormone, *DST* dexamethasone suppression test, *EAS* ectopic adrenocorticotropic syndrome, *SCD* subclinical Cushing’s disease

**Fig. 2:**
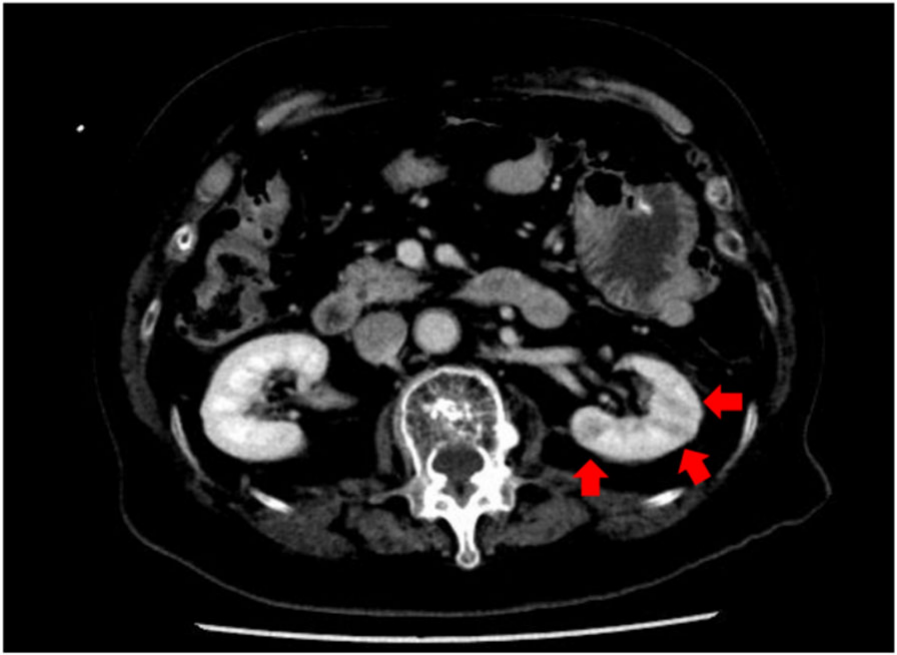
Abdominal computed tomography (CT) on admission. Poor contrast of the left kidney on enhanced CT demonstrating pyelonephritis. red arrows: poor contrast area

In May 2018, she was taken to our hospital because of decreased consciousness. Her body temperature was 37.7℃, pulse rate was 90 beats per minute, and blood pressure was 114/71 mmHg. She had a fever, but her physical examination was unremarkable. Laboratory tests showed a white blood cell (WBC) count of 35,900 /µL, C-reactive protein (CRP) level of 4.3 mg/dL, HbA1c level of 7.9 %, ACTH level of 300.7 pg/mL (normal range: 7.2–63.3 pg/mL) and cortisol level of 22.9 µg/dL (normal range: 4.0–23.3 µg/dL) (Table 2). Enhanced computed tomography showed a poor contrast of the left kidney (Fig. 2), and bacterial cultures from blood and urine were positive for *Escherichia coli*, demonstrating acute pyelonephritis. With antibiotics (meropenem 2 g/day), her fever gradually resolved and the CRP level decreased, while her glycemic control was poor despite intensive insulin therapy (casual blood glucose level was 200 to 300 mg/dL).

**Table 2:** Laboratory findings on admission

Complete blood cell count	
WBC (/µL)	35,900
Neutrophil (%)	96
Lymphocytes (%)	1.5
RBC (/µL)	4,290,000
Hb (g/dL)	12.5
Plt (/µL)	114,000
Biochemistry	
TP/Alb (g/dL)	4.9/2.5
T-Bil (mg/dL)	1.0
AST/ALT/ALP (U/L)	50/22/169
BUN/Cr (mg/dL)	21/0.66
Na/K/Cl (mEq/L)	143/2.2/97
CRP (mg/dL)	4.3
FPG (mg/dL)	111
HbA1c (%)	7.9
Endocrine	
ACTH (pg/mL)	300.7
cortisol (µg/dL)	22.5

*WBC* white blood cell, *RBC* red blood cell, *Hb* hemoglobin, *Plt* platelet, *TP* total protein, *Alb* albumin, *T-Bil* total bilirubin, *AST* aspartate amino transferase, *ALT* alanine amino transferase, *ALP* alkaline phosphatase, *BUN* blood urea nitrogen, *Cr* creatinine, *CRP* C-reactive protein, *FPG* fasting plasma glucose, *HbA1c* hemoglobin A1c, *ACTH* adrenocorticotropic hormone

**Fig. 3:**
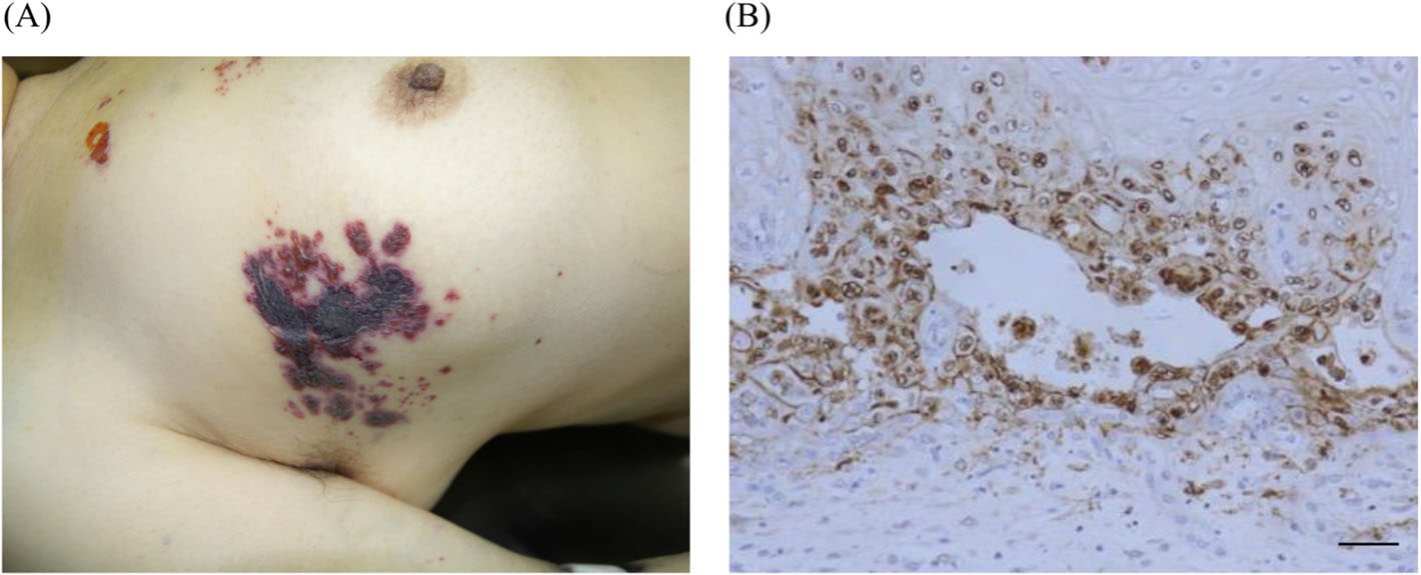
Physical and histological findings demonstrating varicella zoster virus (VZV) infection. **a** Erythematous vesicles appeared on the patient’s chest. **b** VZV immunostaining demonstrating disseminated herpes zoster esophagitis (×200), Scale bar: 50 µm

**Fig. 4:**
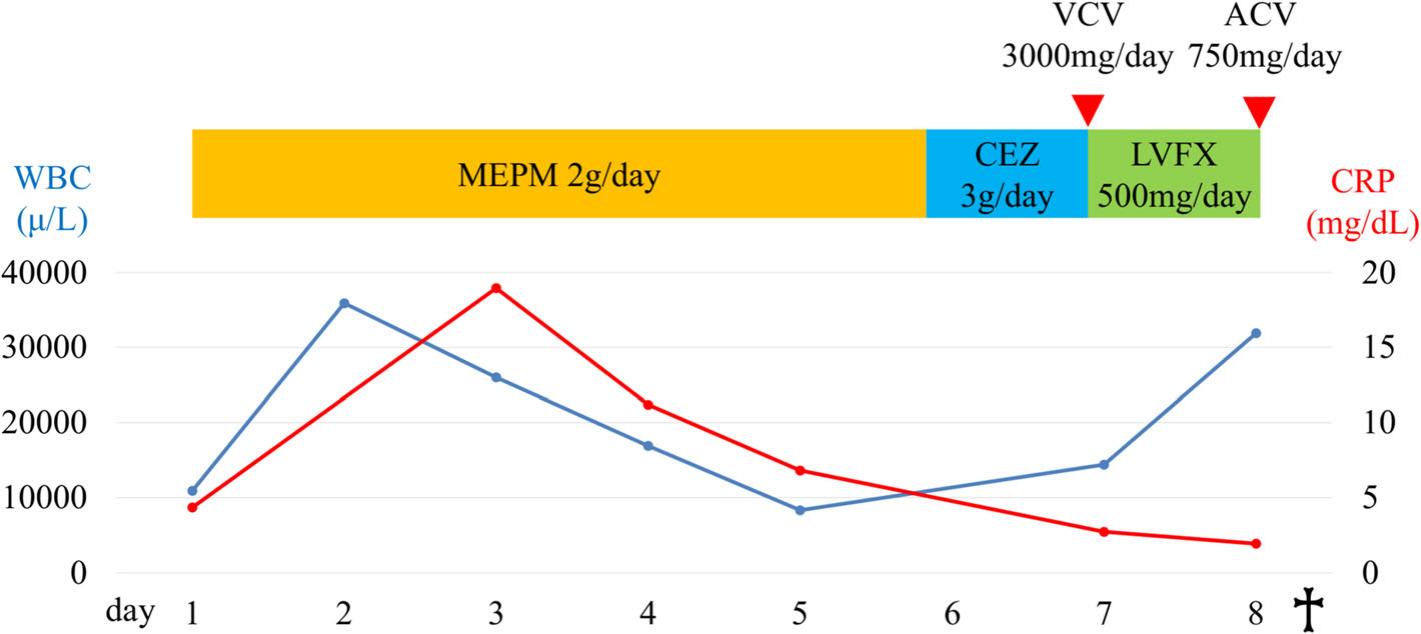
Clinical course after admission. blue line: WBC, white blood cell, led line: CRP, C-reactive protein. MEPM, meropenem; CEZ, cefazolin; LVFX, levofloxacin; VCV, valacyclovir; ACV, acyclovir

Unexpectedly, erythematous vesicles appeared on her chest on the seventh day (Fig. 3a). As skin eruptions rapidly spread over the entire body on the eighth day, we diagnosed the patient with herpes zoster, and started administering antiviral drugs (valacyclovir 3000 mg/day). She did not take steroids or any immunosuppressants. Laboratory tests showed an elevated WBC level, coagulation disorders, liver enzyme elevation and acute kidney injury, suggesting multiple organ insufficiency (Table 3). Human immunodeficiency virus (HIV) antibody was negative. She suddenly went into cardiopulmonary arrest because of circulatory failure caused by septic shock and died despite all our efforts to save her life in the intensive care unit (Fig. 4). As a result of autopsy, the pituitary tumor was ACTH-positive, and other anterior pituitary hormones were negative on immunohistochemical staining. There were no malignant tumors suspected of ectopic ACTH-producing tumor. In addition, autopsy demonstrated renal abscess and varicella zoster virus (VZV) esophagitis (Fig. 3b), suggesting DHZ.

**Table 3:** Laboratory findings when skin eruptions spread over the body (day 8)

Complete blood cell count	
WBC (/µL)	31,900
seg. (%)	50
stab. (%)	28
Lymphocytes (%)	3.5
RBC (/µL)	4,770,000
Hb (g/dL)	14.0
Plt (/µL)	88,000
Coagulation	
PT-INR	4.65
APTT (sec)	70.1
Fib (mg/dL)	<50
D-dimer (μg/mL)	750
Biochemistry	
TP/Alb (g/dL)	5.4/2.5
T-Bil (mg/dL)	2.1
AST/ALT/ALP (U/L)	2953/742/855
BUN/Cr (mg/dL)	27/1.2
Na/K/Cl (mEq/L)	133/3.4/91
CRP (mg/dL)	1.9

*WBC* white blood cell, *seg.* segmented cell, *stab.* stab cell, *RBC* red blood cell, *Hb* hemoglobin, *Plt* platelet, *PT-INR* prothrombin time international normalized ration, *APTT* activated partial thromboplastin time, *Fib* fibrinogen, *TP* total protein, *Alb* albumin, *T-Bil* total bilirubin, *AST* aspartate amino transferase, *ALT* alanine amino transferase, *ALP* alkaline phosphatase, *BUN* blood urea nitrogen, *Cr* creatinine, *CRP* C-reactive protein

## Discussion and conclusions

To the best of our knowledge, this is the first report in which SCD might be one of the triggers of severe infection including DHZ. SCD could cause immunosuppression despite mild hypercortisolism, leading to renal abscess followed by sudden onset of DHZ. These severe infections induced septic shock and multiple organ failure, ultimately resulted in mortal outcome.

Infection is one of the major causes of death in patients with CD. A previous report has shown that 28/311 (9 %) of patients with CD that were followed up for a median period of 9 years died from cardiovascular causes in 14/28 (50 %), infection in 6/28 (21.4 %), and malignancy in 7/28 (25 %) [8]. Although no reports on the mortality of SCD are available, adrenal adenomas with mild hypercortisolism cause infection-related mortal outcomes [9]. We may need to pay attention to infection in patients with SCD as well as patients with CD.

DHZ infection often invades other organs, and occurs in patients with immunosuppression [10]. Immunosuppression caused by malignancy, HIV infection, and immunosuppressive therapy such as steroids increases the risk for herpes zoster infection [11]. Also, aging and diabetes mellitus were reported to increase the risk of herpes zoster infection [12]. In this case, hypercortisolism caused by SCD made it difficult to maintain normal blood glucose level. Although malignancy, HIV infection, and history of immunosuppressive therapy were not observed in this case, aging and secondary diabetes mellitus caused by SCD might be associated with the onset of DHZ.

On the other hand, infection in CD is a consequence of the immunosuppression induced by hypercortisolism [6]. In addition, disorders of circadian cortisol rhythm are associated with immunosuppression. Excess and dysrhythmic cortisol exposure can lead neutrophil dysfunction, decreased lymphocytes, and inactivation of lymphocytes, resulting in impaired immune function [9, 13]. In this case, the plasma cortisol level remained high at night-time, suggesting disappearance of the circadian rhythm of cortisol and lymphocytes. Furthermore, laboratory tests before admission showed that lymphocyte counts and IgG level were decreased (Supplementary Table 1), suggesting impaired immune function caused by SCD. As well as aging or diabetes, mild hypercortisolism and dysrhythmic cortisol exposure caused by SCD can be one of the triggers of severe infections including DHZ.

In conclusion, we reported a case in which SCD might be one of the triggers of severe infection including DHZ. As our case emphasized the risk of severe infections in the patients with SCD, the patients should be assessed not only for the metabolic but also for the immunodeficient status.

## Supplementary information

Additional file 1 **Supplementary Table 1.** Laboratory data before admission

## Data Availability

All data from this article are available from the corresponding authors upon request.

## Abbreviations

ACTH: Adrenocorticotropic hormone
CD: Cushing’s disease
CRP: C-reactive protein
CS: Cushing’s syndrome
DHZ: Disseminated herpes zoster
DST: Dexamethasone suppression test
HIV: Human immunodeficiency virus
MRI: Magnetic resonance imaging
SCD: Subclinical Cushing’s disease
VZV: Varicella zoster virus
WBC: White blood cell
