# Supplementary material for: Severe infection including disseminated herpes zoster triggered by subclinical Cushing’s disease: a case report
Source: BMC Endocr Disord. 2021 Apr 27;21:84. doi: 10.1186/s12902-021-00757-y (PMC8077829; doi:10.1186/s12902-021-00757-y)
Supplement: Supplementary file 1 — Supplementary Table 1. Laboratory data before admission [file 12902_2021_757_MOESM1_ESM.docx]

Supplementary Table 1. Laboratory data before admission

| Complete blood cell count |  |
| --- | --- |
| WBC (/μL) | 6700 |
| Neutrophil (%) | 88 |
| Lymphocytes (%)  Lymphocyte counts | 6.0  402 |
| RBC (/μL) | 5090000 |
| Hb (g/dL) | 15.7 |
| Plt (/μL) | 149000 |
| Biochemistry  TP/Alb (g/dL)  T-Bil (mg/dL)  AST/ALT/ALP (U/L)  BUN/Cr (mg/dL)  Na/K/Cl (mEq/L) | 6.8/3.9  1.4  38/55/257  15/0.49  141/3.3/99 |
| IgG (mg/dL)  IgM (mg/dL)  IgA (mg/dL) | 710  117  367 |
| Endocrine |  |
| ACTH (pg/mL) | 169.8 |
| cortisol (μg/dL) | 29.6 |

WBC, white blood cell; RBC, red blood cell; Hb, hemoglobin; Plt, platelet; TP, total protein; Alb, albumin; T-Bil, total bilirubin; AST, aspartate amino transferase; ALT, alanine amino transferase; ALP, alkaline phosphatase; BUN, blood urea nitrogen; Cr, creatinine; IgG, immunoglobulin G; IgM, immunoglobulin M; IgA, immunoglobulin A; ACTH, adrenocorticotropic hormone.
